# DNA damage response and repair gene mutations are associated with tumor mutational burden and outcomes to platinum-based chemotherapy/immunotherapy in advanced NSCLC patients

**DOI:** 10.1186/s13000-023-01401-0

**Published:** 2023-11-03

**Authors:** Weiguang Gu, Wenya Zhuang, Mengxia Zhuang, Minhong He, Zhihua Li

**Affiliations:** 1 Department of Oncology, Nanhai People’s hospital/the Sixth Affiliated Hospital of South China University of Technology, Foshan, 528200 Guangdong province China; 2 The Second Clinical Medical College, Southern Medical University, Guangzhou, 510515 Guangdong province China

**Keywords:** DDR gene, Non-small cell Lung cancer, Platinum-based chemotherapy, Platinum-based chemotherapy/immunotherapy, Metastasis

## Abstract

**Background:**

DNA damage response and repair (DDR) genes are crucial for maintaining the integrity of the genome. This study aims to explore the correlation of DDR gene mutations with TMB, clinical characteristics, and outcomes to platinum-based chemotherapy and platinum-based chemotherapy/immunotherapy in non-small cell lung cancer (NSCLC) without *EGFR* and *ALK* alterations.

**Methods:**

Tumor tissue from 49 patients with stage III or IV NSCLC who were without *EGFR* and *ALK* alterations were analyzed using targeted next-generation sequencing (NGS). Among them, 13 patients received first-line platinum-based chemotherapy, 32 patients received first-line platinum-based chemotherapy/immunotherapy.

**Results:**

In these NSCLC patients without *EGFR* and *ALK* alterations, the frequently mutated genes included *TP53*, *KMT2D* and *KRAS*, the most frequently mutated DDR gene was *FANCG*, DDR gene mutations were detected in 20 patients. The mutation frequency of homologous recombination (HR) pathway was significantly higher in lung squamous cell carcinoma (LUSC) than that in lung adenocarcinoma (LUAD) (30.8% vs. 5.7%). Among DDR positive patients, a lower percentage exhibited metastasis. Patients with DDR gene mutations, cell-cycle checkpoint pathway mutations, and BER pathway mutations had significantly higher TMB compared to those without corresponding mutations. In the patients receiving platinum-based chemotherapy/immunotherapy, the disease control rate was significantly lower in the DDR-positive group compared with that in the DDR-negative group (55.6% vs. 100.0%). Among LUAD patients receiving platinum-based chemotherapy/immunotherapy, we observed a worse overall survival (OS) in DDR-positive group, as well as poorer progression-free survival(PFS)and OS in BER-positive and *FANCG* mutated group.

**Conclusions:**

DDR gene mutations are associated with tumor metastasis, TMB, and outcomes to platinum-based chemotherapy/immunotherapy in advanced NSCLC patients.

## Introduction

Lung cancer is the leading cause of death worldwide. In 2020, 2.2 million new cases were diagnosed as lung cancer, and 1.8 million individuals died from lung cancer [1]. Non-small cell lung cancer (NSCLC) accounts for more than 80% of all lung cancer cases [2, 3]. In NSCLC patients with mutations in *ALK* and *EGFR*, the efficacy of tyrosine kinase inhibitor (TKI)-based target therapy has been fully demonstrated [4]. However, a considerable proportion of NSCLC patients lack driver gene variants which can be targeted for effective therapies [5, 6]. Chemotherapy and immunotherapy are considered to be important for these driver gene negative patients [7–9].

DNA damage response and repair (DDR) genes play a key role in detecting and repairing DNA damage, are crucial for maintaining the integrity of the genome [10, 11]. DDR genes were categorized into various functional pathways, including the base excision repair (BER), nucleotide excision repair (NER), mismatch repair (MMR), non-homologous end joining (NHEJ), homologous recombination (HR), cell-cycle checkpoint (CCK) and Fanconi anemia (FA) pathways [10, 12]. Deficiency in DDR genes were reported to be associated with cancer development and responses to varies therapies [13]. DDR gene mutations were shown to be associated with improved clinical outcomes in urothelial carcinoma and ovarian cancer [14, 15]. In non-small cell lung cancer, DDR gene mutations had an impact on response and prognosis for patients received immunotherapy [16]. Nevertheless, there are few studies exploring the DDR gene mutational characteristics and their correlation with outcomes to platinum-based chemotherapy and platinum-based chemotherapy/immunotherapy in NSCLC without *EGFR* and *ALK* alterations.

In this study, we aim to investigate the mutation features of DDR genes, to reveal their associations with clinical characteristics, biomarker, response, and prognosis in advanced NSCLC patients without *EGFR* and *ALK* alterations who received platinum-based chemotherapy and platinum-based chemotherapy/immunotherapy.

## Materials and methods

### Patients and sample collection

Patients with 19del, L858R, T790M, 20ins, G719X (X = A, C, or S), S768I, or L861Q were classified as individuals with *EGFR* alterations. Patients with positive *EML4-ALK* fusion were classified as individuals with *ALK* alterations. Formalin-fixed and paraffin-embedded (FFPE) tumor tissue samples or blood samples from patients with stage III or IV NSCLC were collected and tested by 556 gene panel (Shanghai Tongshu Biotechnology Co., Ltd, Shanghai, China). 49 patients who were without *EGFR* and *ALK* alterations were enrolled in the study. Among these patients, 13 patients received first-line platinum-based chemotherapy, 32 patients received first-line platinum-based chemotherapy and immune checkpoint inhibitors (ICI) therapy (platinum-based chemotherapy/immunotherapy). The clinicopathological data for each patient was collected. This study was conducted in accordance with the Declaration of Helsinki.

### Sample preparation and 556 gene targeted sequencing

We extracted DNA from FFPE tissue samples using Tissue Kit (69,504, QIAGEN, Venlo, the Netherlands) following the manufacturer’s protocols. The ctDNA was extracted from plasma according to the manufacturers’ instructions (HiPure Circulating DNA Midi Kit, Magen, Guangzhou, China). Germline DNA was extracted from blood lymphocytes using HiPure Blood DNA Mini Kit (Magen, Guangzhou, China). Tumor tissue sections were examined by two pathologists independently and only those containing at least 30% tumor cells were selected for further detection. A NadPrep DNA Library Preparation Kit (Nanodigmbio, Nanjing, China) was used to prepare libraries for the Illumina sequencing platform. A NadPrep Hybrid Capture Reagents kit (Nanodigmbio, Nanjing, China) was used for hybridization capture–based target enrichment. The captured libraries sequencing was performed as paired-end reads on the Illumina Novaseq 6000 platform.

### Data processing

The sequence data were filtered by Fastp (version 0.20.0) and aligned to the human reference genome (hg19) using Burrows-Wheeler Aligner (BWA, Version: 0.7.12-r1039). Single nucleotide variants (SNVs) and insertion/deletion (Indel) were detected using vardict-java (version 1.7.0–0) and Mutect2 of GATK4 (version4.1.8.1). Structural variations were detected using factera (version 1.4.4). After filtering, those variants were then annotated by ensembl’s Variant Effect Predictor (VEP, version 104).

### Statistical analysis

Fisher’s exact or chi-square test was applied to compare the categorical variables between the groups. All reported *P* values were two-sided, and *P* < 0.05 was considered statistically significant.

## Results

### Demographic and clinicopathological information in the study cohort

49 patients diagnosed with stage III or IV NSCLC who were without *EGFR* and *ALK* alterations were enrolled in the study. The clinical characteristics of these patients were summarized in Table 1. The age of the cohort ranged from 47 to 77 years (median: 62). The majority of the patients were males (69.4%). Most of the tumors were adenocarcinoma (71.4%) and categorized as stage IV (83.7%). Among these patients, 13 patients received first-line platinum-based chemotherapy, 32 patients received first-line platinum-based chemotherapy in combination with immunotherapy.

**Table 1:** Patient demographics and clinical characteristics

Clinical Characteristics	All (n = 49)
**Age (Median = 62)**	
<65	33 (67.3%)
≥ 65	16 (32.7%)
**Gender**	
Male	34 (69.4%)
Female	15 (30.6%)
**Smoking status**	
Smoker	23 (46.9%)
Non-smoker	26 (53.1%)
**Stage**	
III	8 (16.3%)
IV	41 (83.7%)
**Metastasis**	
0	8 (16.3%)
1	19 (38.8%)
> 1	22 (44.9%)
**NSCLC subtype**	
Adenocarcinoma	35 (71.4%)
Squamous	13 (26.5%)
other	1 (2.0%)
**Response**	
CR	1 (2.0%)
PR	21 (42.9%)
SD	19 (38.8%)
PD	6 (12.2%)
NA	2 (4.1%)
**Therapy**	
Platinum-based chemotherapy	13 (26.5%)
Platinum-based chemotherapy/immunotherapy	32 (63.3%)

### The mutational landscape of advanced NSCLC without *EGFR* or *ALK* gene alterations

**Fig. 1:**
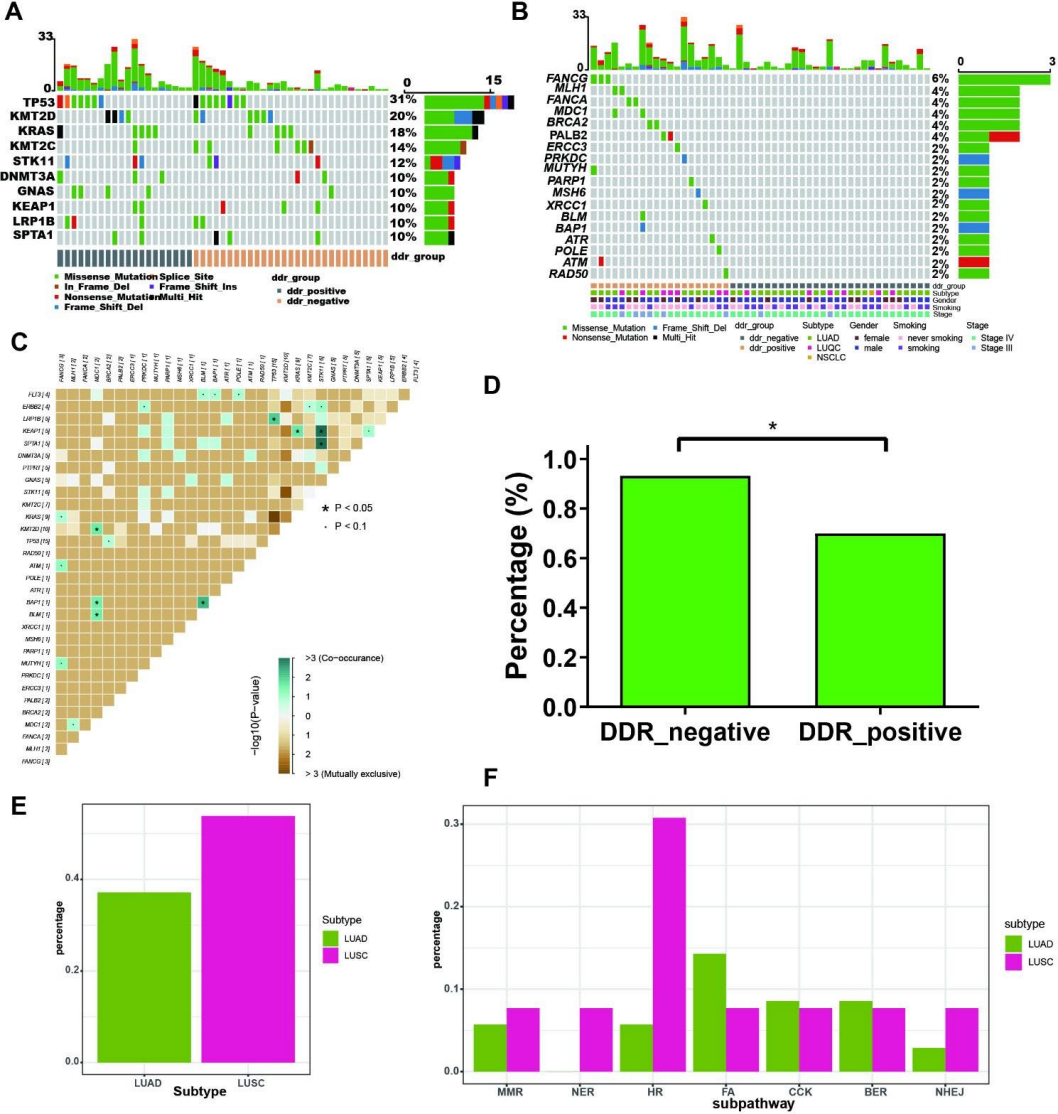
The mutational landscape of advanced NSCLC without *EGFR* or *ALK* gene alterations. (**A**) The frequently mutated genes of advanced NSCLC without *EGFR* or *ALK* gene alterations. (**B**) The mutated DDR genes and clinical features of advanced NSCLC without *EGFR* or *ALK* gene alterations. (**C**) The interaction between DDR genes and frequently mutated genes. (**D**) The percentage of patients with metastasis in DDR negative and DDR positive patients. (**E**) The percentage of DDR positive patients in LUAD and LUSC. (**F**) The percentage of MMR-positive, NER-positive, HR-positive, FA-positive, CCK-positive, BER -positive, and NHEJ-positive patients in LUAD and LUSC.

Among these advanced NSCLC without *EGFR* or *ALK* gene alterations, the frequently mutated genes included *TP53*, *KMT2D* and *KRAS* (Fig. 1A). DDR gene mutations were observed in 20 patients (40.8%). Among these patients with DDR gene mutations, 80.0% patients had mutations in only one DDR gene, 4 (20.0%) patients had mutations in more than one DDR gene (Fig. 1B). *FANCG* was the most frequently mutated DDR gene. Furthermore, the interaction among frequently mutated genes and DDR genes was explored. We observed co-occurrences between DDR genes and frequently mutated genes, as well as between different frequently mutated genes. Mutations in the DDR gene *MDC1* was observed co-occurring with mutations in frequently mutated *KMT2D* gene. The frequently mutated *TP53* gene was observed co-occurring with the frequently mutated *LRP1B* gene. The frequently mutated *KEAP1* was observed co-occurring with the frequently mutated *KRAS* and *STK11* (Fig. 1C).

Then, the correlation between clinical characteristics and DDR mutation was explored. Among DDR positive patients, there was a lower percentage of individuals with metastasis (Table 2; Fig. 1D). There were no significant differences in age, gender, smoking history, and tumor size between the DDR-positive and DDR-negative groups (Table 2). In addition, we observed that different histopathological subtypes exhibited specific mutation characteristics in the DDR pathways. The mutation frequency of all DDR genes had a trend to be higher in lung squamous cell carcinoma (LUSC) (53.8%) than that in lung adenocarcinoma (LUAD) (37.1%), with no significant difference (Fig. 1E). The mutation frequency of HR pathway was significantly higher in LUSC (30.8%) compared to LUAD (5.7%) (Fig. 1F).

**Table 2:** Association between clinical characteristics and DDR gene mutations in advanced NSCLC patients

	Negative	Positive	Odd ratio	P value
**Age**				
< 65	20	13	1.19	1
>=65	9	7		
**Gender**				
female	8	7	0.71	0.75
male	21	13		
**Smoking history**				
Never_smoking	13	13	0.45	0.24
Smoking	16	7		
**Tumor size**				
Small	12	12	0.48	0.25
Big	17	8		
**Metastasis**				
Never_metastasis	2	6	0.18	0.0497
Metastasis	27	14		
**Therapy**				
Platinum-based chemotherapy/immunotherapy	21	11	2.97	0.11
Platinum-based chemotherapy	5	8		

### Increased TMB observed in NSCLC patients with DDR mutations, cell-cycle checkpoint pathway mutations, and BER pathway mutations

**Fig. 2:**
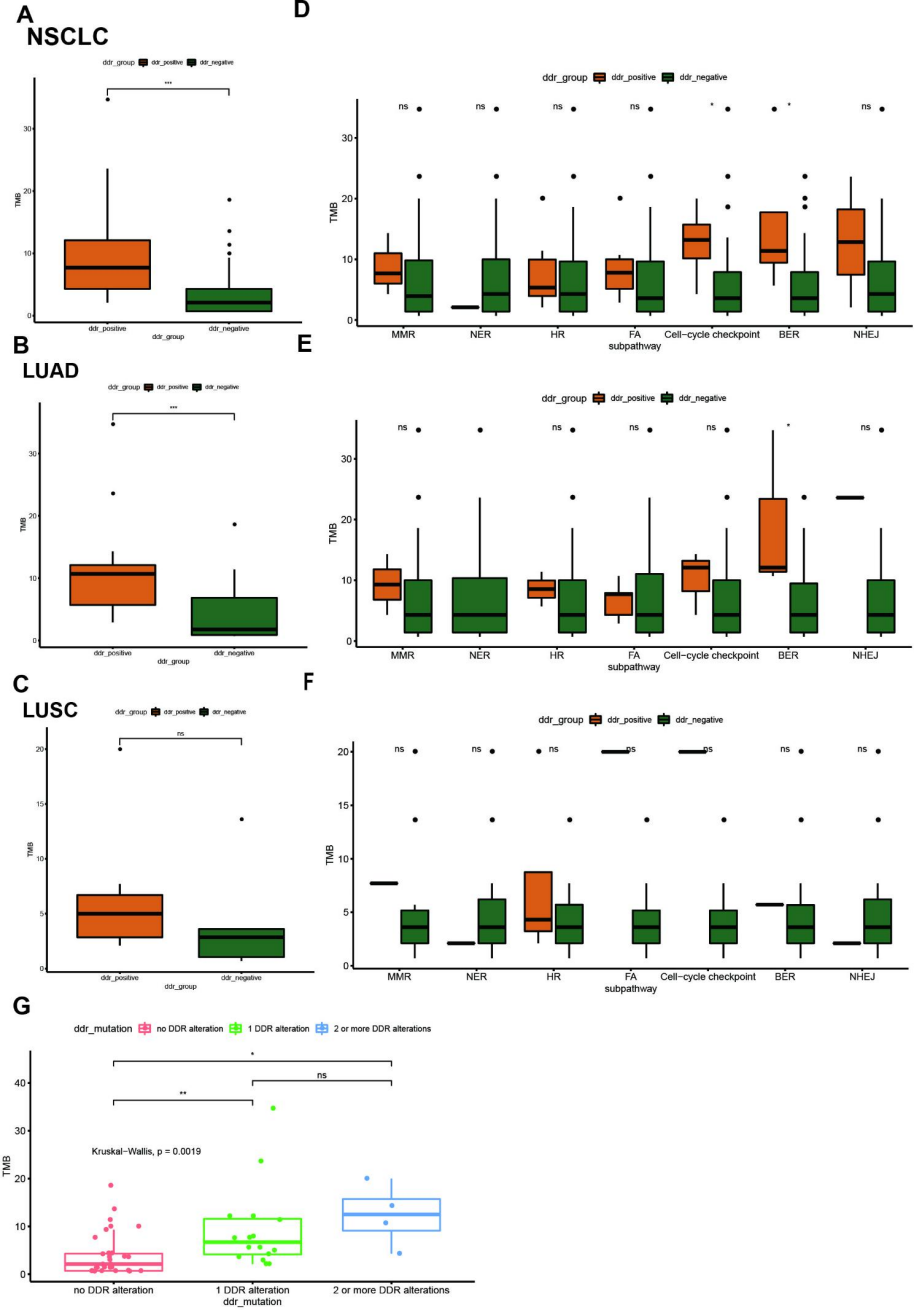
Relationships between DDR gene mutations and TMB in NSCLC patients without *EGFR* or *ALK* gene alterations. (**A-C**) Relationship between DDR gene mutations and TMB in all NSCLC patients, LUAD patients and LUSC patients. (**D-F**) Relationship between mutations of different DDR pathways and TMB in all NSCLC patients, LUAD patients and LUSC patients. (**G**) Relationship between the number of DDR gene mutations and TMB in all NSCLC patients. LUAD: lung adenocarcinoma, LUSC: lung squamous cell carcinoma

TMB was compared between DDR-positive and DDR-negative patients. The median TMB value was higher in patients with DDR gene mutations (7.7 vs. 2.1 mut/Mb). Furthermore, TMB was compared in patients with mutations in each DDR pathway. Patients with cell-cycle checkpoint pathway mutations and BER pathway mutations had significantly higher TMB than those without corresponding mutations (Fig. 2A and D). The analysis in the LUAD cohort showed that significantly higher TMB was observed in NSCLC patients with DDR mutations and BER pathway mutations. Patients with cell-cycle checkpoint pathway mutations also showed higher TMB in the LUAD cohort, although the difference was not statistically significant (Fig. 2B and E). The analysis in the LUSC cohort showed that the TMB had a trend to be higher in DDR mutated patients, although no significant difference was observed due to the limited number of patients (Fig. 2C F). TMB had a trend to be increased with the number of DDR alterations (Fig. 2G). Thus, among the DDR pathway, cell-cycle checkpoint pathway and BER pathway were significant associated with higher TMB in advanced NSCLC patients.

### Association between DDR mutation status and clinical responses to platinum-based chemotherapy and platinum-based chemotherapy/immunotherapy in NSCLC patients

**Fig. 3:**
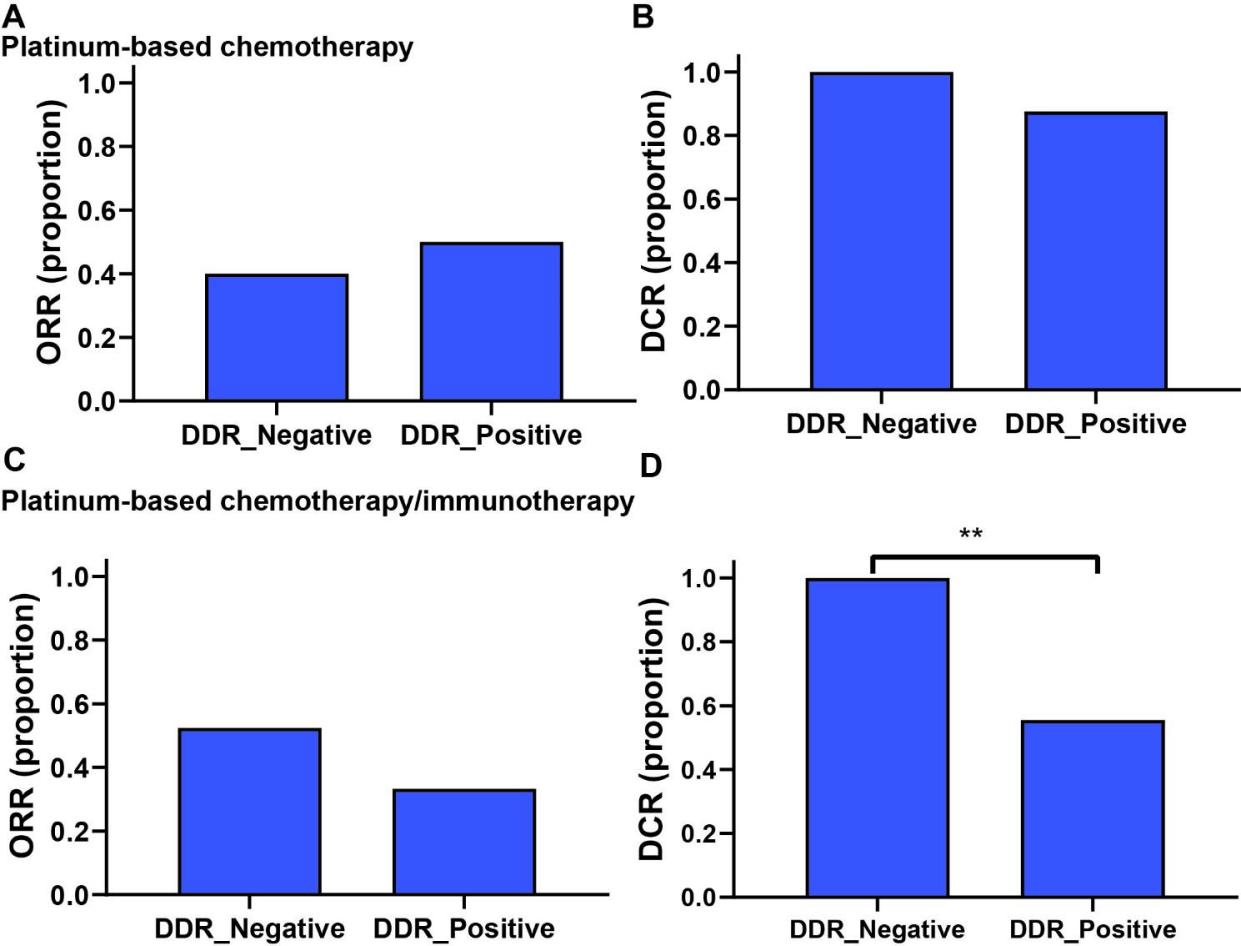
Association between DDR gene mutations and clinical outcomes to platinum-based chemotherapy and platinum-based chemotherapy/immunotherapy. (**A**) Association between DDR mutations and ORR in patients receiving platinum-based chemotherapy. (**B**) Association between DDR mutations and DCR in patients receiving platinum-based chemotherapy. (**C**) Association between DDR mutations and ORR in patients receiving platinum-based chemotherapy/immunotherapy. (**D**) Association between DDR mutations and DCR in patients receiving platinum-based chemotherapy/immunotherapy. ORR: objective response rate, DCR: disease control rate

We next compared clinical responses to platinum-based chemotherapy and platinum-based chemotherapy/immunotherapy according to DDR mutation status. In the patients receiving platinum-based chemotherapy, the objective response rate (ORR) was 46.2%, and the disease control rate (DCR) was 92.3%. The ORR and DCR were similar in DDR-positive and DDR-negative group (Fig. 3A and B; Table 3). In the patients receiving platinum-based chemotherapy/immunotherapy, the ORR was 46.7%, the DCR was 86.7%. The ORR showed no significant difference in DDR-positive and DDR-negative group (Fig. 3C). While the DCR was shown to be significantly lower in the DDR-positive group compared with that in the DDR-negative group (55.6% vs. 100.0%) (Fig. 3D; Table 3).

**Table 3:** Association between DDR gene nutations and clinical responses to platinum-based chemotherapy and platinum-based chemotherapy/immunotherapy in advanced NSCLC patients

Platinum-based chemotherapy				
Response	DDR negative	DDR positive	Odd ratio	P value
**ORR**			1.45	1
**non-ORR**	3	4		
**ORR (CR + PR)**	2	4		
**DCR**			0	1
**non-DCR**	0	1		
**DCR (CR + PR + SD)**	5	7		
**Platinum-based chemotherapy/immunotherapy**				
**Response**	DDR negative	DDR positive	Odd ratio	P value
**ORR**			0.47	0.44
**non-ORR**	10	6		
**ORR (CR + PR)**	11	3		
**DCR**			0	0.0046
**non-DCR**	0	4		
**DCR (CR + PR + SD)**	21	5		

### The impact of DDR mutations on prognosis in NSCLC patients receiving platinum-based chemotherapy and platinum-based chemotherapy/immunotherapy

**Fig. 4:**
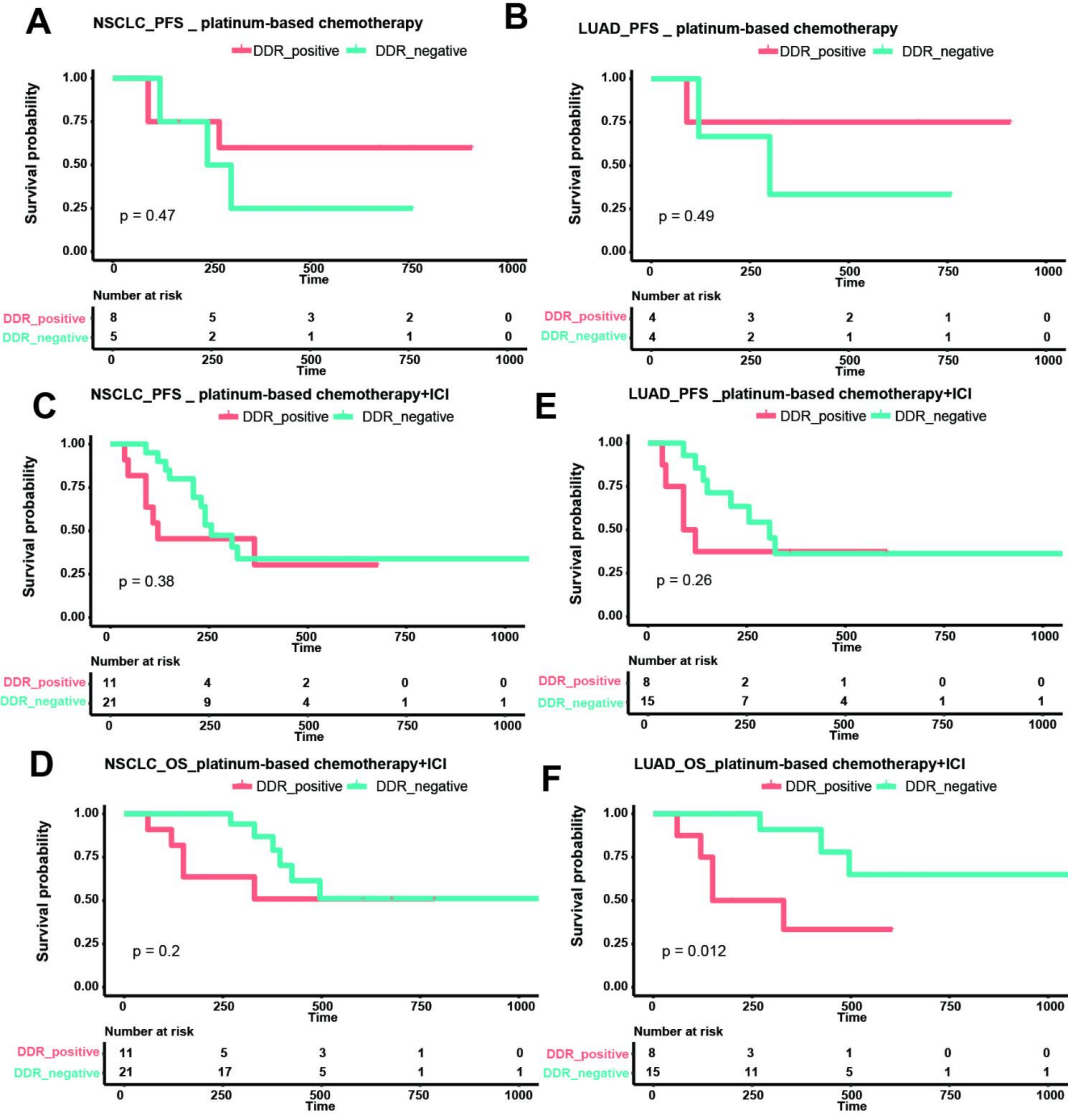
Kaplan–Meier survival analysis for PFS and OS according to DDR gene mutation status. (**A-B**) Survival analysis of DDR mutations on the PFS in all NSCLC patients receiving platinum-based chemotherapy (**A**) and LUAD patients receiving platinum-based chemotherapy (**B**). (**C-F**) Survival analysis of DDR mutations on the PFS and OS in all NSCLC patients receiving platinum-based chemotherapy/immunotherapy (**C-D**) and LUAD patients receiving platinum-based chemotherapy/immunotherapy (**E-F**). PFS: progression-free survival, OS: overall survival

The impact of DDR mutations on prognosis in NSCLC patients receiving platinum-based chemotherapy and platinum-based chemotherapy/immunotherapy was explored. The progression-free survival (PFS) and overall survival (OS) were compared in DDR-positive and DDR-negative patients (Fig. 4). Among all NSCLC patients receiving platinum-based chemotherapy, DDR-positive patients showed a trend towards better PFS, with no significant difference (Fig. 4A). A similar trend was observed in LUAD patients receiving platinum-based chemotherapy (Fig. 4B). The OS in patients who received platinum-based chemotherapy was not compared, since all these patients were alive at the time of analysis. In all NSCLC patients who received platinum-based chemotherapy/immunotherapy, no significant difference in PFS and OS was observed between DDR-positive and DDR-negative group (Fig. 4C and D). While in LUAD patients receiving platinum-based chemotherapy/immunotherapy, a worse OS was observed in DDR-positive group (Fig. 4E and F).

**Fig. 5:**
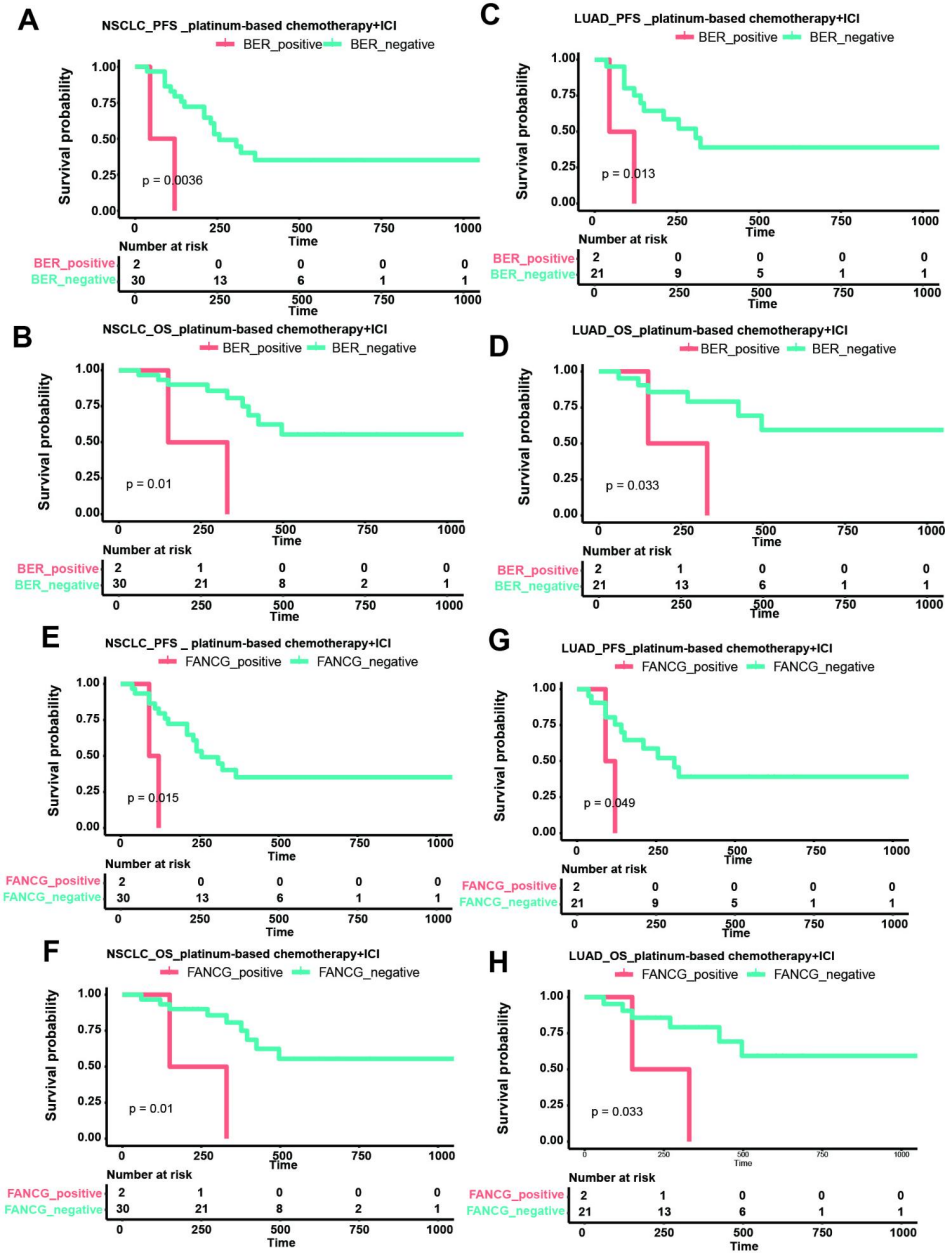
Kaplan–Meier survival analysis for PFS and OS according to mutation status in BER pathway and *FANCG* gene. (**A-D**) Survival analysis of BER pathway mutations on the PFS and OS in all NSCLC patients receiving platinum-based chemotherapy/immunotherapy (**A-B**) and LUAD patients receiving platinum-based chemotherapy/immunotherapy (**C-D**). (**E-H**) Survival analysis of *FANCG* mutations on the PFS and OS in all NSCLC patients receiving platinum-based chemotherapy/immunotherapy (**E-F**) and LUAD patients receiving platinum-based chemotherapy/immunotherapy (**G-H**). PFS: progression-free survival, OS: overall survival

Furthermore, the impact of different DDR pathways and DDR genes on prognosis was investigated (Fig. 5). In all NSCLC patients receiving platinum-based chemotherapy/immunotherapy, poorer PFS and OS were observed in patients with BER pathway mutations and those with *FANCG* mutations (Fig. 5A, B, E, and F). Similarly, in LUAD patients receiving platinum-based chemotherapy/immunotherapy, significantly poorer PFS and OS were also observed in patients with BER pathway mutations and those with *FANCG* mutations (Fig. 5C, D, G, and H).

### The impact of DDR mutations on TMB and Tumor Metastasis in TCGA cohort

**Fig. 6:**
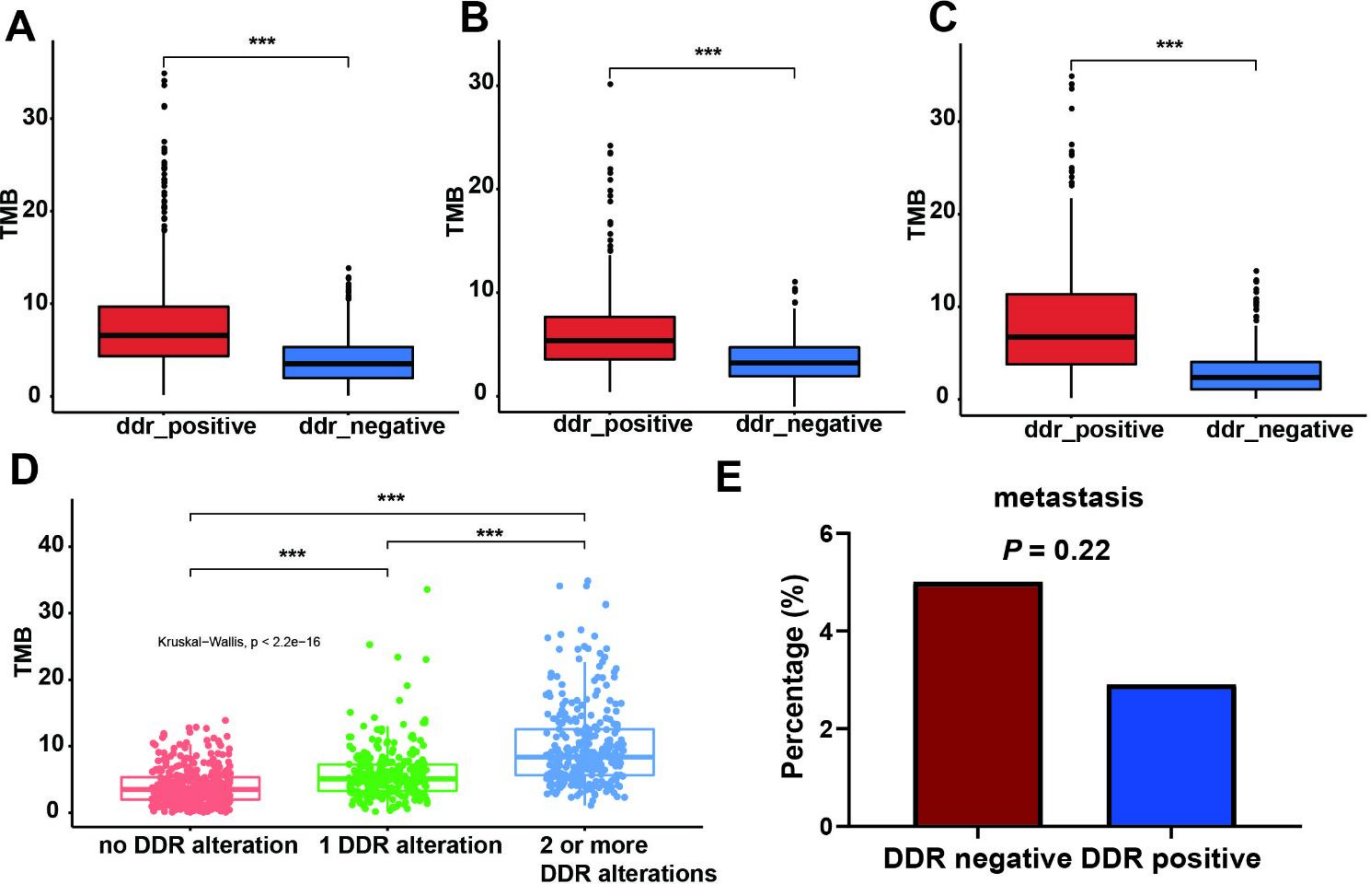
Relationships of DDR gene mutations, TMB and metastasis in NSCLC patients without *EGFR* and *ALK* alterations from TCGA cohort. (**A-C**) Relationship between DDR gene mutations and TMB in all NSCLC (**A**), LUAD (**B**) and LUSC (**C**). (**D**) Relationship between the number of DDR gene mutations and TMB. (**E**) Relationship between DDR gene mutations and metastasis

Then, the correlation between DDR mutations, TMB and tumor metastasis was explored in TCGA cohort. The significantly higher TMB was observed in DDR positive cohort from LUAD and LUSC cohort (Fig. 6A, B, and C). In addition, TMB increased with the number of DDR alterations (Fig. 6D). DDR positive cohort exhibited a tendency towards lower metastasis rates, although no significant difference was observed (Fig. 6E). These findings regarding the impact of DDR mutations on TMB and tumor metastasis in TCGA cohort was similar with the results obtained from the samples examined in this study.

## Discussion

To our knowledge, this is the first systematic study for the impact of DDR gene mutations on prognosis and response to platinum-based chemotherapy and platinum-based chemotherapy/immunotherapy in NSCLC patients without *EGFR* and *ALK* alterations. Although the efficacy of TKI-based target therapy has been demonstrated in patients with *EGFR* and *ALK* alterations, there remains a need for prognostic indicators for those without these driver gene mutations who received chemotherapy and immunotherapy. By conducting next generation sequencing, the study revealed the mutational landscape and the clinical value of DDR gene mutations in predicating tumor metastasis and outcomes to platinum-based chemotherapy and platinum-based chemotherapy/immunotherapy in NSCLC patients without *EGFR* and *ALK* alterations. In NSCLC patients received platinum-based chemotherapy, DDR-positive patients trended to have a better prognosis, with no significant difference. In the patients received platinum-based chemotherapy/immunotherapy, poor prognosis was observed in DDR-positive patients.

Our study showed that the most frequently mutated genes included *TP53*, *KMT2D* and *KRAS* in NSCLC patients without *EGFR* and *ALK* mutations. In similar with our results, *TP53* and *KRAS* were also reported to be frequently mutated genes by Yayi He et al. [17]. In consistent with previous reports [10, 16], DDR positive patients showed higher TMB. DDR related pathway analysis in our study indicated that cell-cycle checkpoint pathway and BER pathway played a key role of the higher TMB in NSCLC patients without *EGFR* and *ALK* alterations. Furthermore, the observed increase in TMB with the number of DDR alterations in our study was supported by Biagio Ricciuti et al’s study [16].

DDR positive patients exhibited a lower percentage of metastasis in our study. The similar trend was observed in TCGA cohort, although the difference was not significant, probably due to the limited number of metastatic individuals. Only 3.7% patients with metastasis were in the TCGA cohort. In support of our results, visceral metastasis showed an inverse trend with an increasing number of DDR alterations in urothelial carcinoma [14]. Therefore, it is essential to pay greater attention to metastasis risk in DDR-negative NSCLC patients.

Some studies regarding to DDR gene and clinical outcomes in lung cancer have been reported. For instance, RANBP9 is a mediator of cellular DNA damage response in lung cancer cells [18]. A retrospective analysis involving 132 patients receiving platinum therapy indicated that RANBP9 overexpression was associated with clinical response to platinum therapy. *RANBP9* KO NSCLC cells was shown to have higher sensitivity to cisplatin compared to WT controls [19]. *PALB2* is a gene in the homologous recombination repair (HRR) pathway. Jiexia Zhang et al. showed that it may not be a promising prognosis predicator on immunotherapy [20]. DDR pathway alterations in SCLC had limited predictive value for platinum efficacy [21]. DDR mutations were shown to be associated with clinical outcomes to immunotherapy in NSCLC patients [16]. Nonetheless, the studies regarding to DDR genes and clinical outcomes in NSCLC receiving platinum-based chemotherapy and platinum-based chemotherapy/immunotherapy are very limited and the results remained confused. Thus, more studies are needed for a clearer understanding.

Chemotherapy and immunotherapy are considered to be important for these driver gene negative patients who were without *EGFR* and *ALK* alterations. Platinum-based chemotherapy is the standard-of-care for most advanced NSCLC patients [22]. We systematically explored the correlation of DDR gene mutations and outcomes to platinum-based chemotherapy and platinum-based chemotherapy/immunotherapy. For patients received platinum-based chemotherapy, DDR-positive patients trended to have better PFS. DDR mutations were also reported to be associated with improved clinical outcomes to platinum-based chemotherapy in urothelial carcinoma, pancreatic cancer, and prostate cancer [14, 23, 24]. Thus, further research with a larger patient cohort is warranted to more comprehensively explore the impact of DDR mutations on outcomes to platinum-based chemotherapy in NSCLC. For LUAD patients received platinum-based chemotherapy/immunotherapy, poor OS was observed in DDR-positive group, poor PFS and OS were observed in BER-positive and *FANCG* mutated group. While in all NSCLC patients receiving platinum-based chemotherapy/immunotherapy, no significantly different PFS and OS were observed in DDR-positive and DDR-negative group. This discrepancy may be associated with the inclusion of LUSC samples in all NSCLC analysis. BER is a DNA repair pathway which is associated with the maintenance of genome stability and cancer. FANCG is a member of FA pathway which is a unique DNA damage repair pathway [25]. The cause for this correlation of these DDR gene mutations and outcomes to platinum-based chemotherapy/immunotherapy need further exploration.

Our study has several limitations that should be acknowledged. Firstly, a longer follow-up period may be required to better assess the impact of DDR gene mutations on the outcomes of first-line platinum-based chemotherapy and first-line platinum-based chemotherapy/immunotherapy in advanced NSCLC patients. Secondly, in our study, 13 patients received first-line platinum-based chemotherapy, and 32 patients received first-line platinum-based chemotherapy/immunotherapy. Research with a larger sample size may be needed to further evaluate the influence of DDR mutations on the outcomes of first-line platinum-based chemotherapy and first-line platinum-based chemotherapy/immunotherapy furthermore in advanced NSCLC patients.

In conclusion, DDR gene mutations are associated with tumor metastasis, TMB, and outcomes to platinum-based chemotherapy/immunotherapy in NSCLC patients. To our knowledge, this is the first systematic study investigating the impact of DDR gene mutations on prognosis and response to platinum-based chemotherapy and platinum-based chemotherapy/immunotherapy in NSCLC patients without *EGFR* and *ALK* alterations. DDR gene mutations hold promise as potential prognosis predicators for NSCLC patients receiving platinum-based chemotherapy/immunotherapy.

## Data Availability

All data generated or analyzed during this study are included in this published article.

## Abbreviations

DDR: DNA damage response and repair
NGS: Next-generation sequencing
OS: Overall survival
PFS: Progression-free survival
LUAD: Lung adenocarcinoma
LUSC: Lung squamous cell carcinoma
ORR: Objective response rate
DCR: Disease control rate
TMB: Tumor mutation burden
